# Validation of whole genome sequencing from dried blood spots

**DOI:** 10.1186/s12920-021-00951-w

**Published:** 2021-04-20

**Authors:** Pooja Agrawal, Shanmukh Katragadda, Arun K. Hariharan, Vijayashree Gauribidanur Raghavendrachar, Arunika Agarwal, Rashmi Dayalu, Disha Awasthy, Sanjay C. Sharma, Yasodha Kannan Sivasamy, P. Lakshmana, Ashwini Shanmugam, Vamsi Veeramachaneni, Vaijayanti Gupta, B. P. Vani, Lekha Subaiya, T. S. Syamala, Ramesh Hariharan, Vijay Chandru, David E. Bloom

**Affiliations:** 1 Strand Life Sciences Pvt. Ltd., Ground Floor, UAS Alumni Association Building, Veterinary College Campus, Bellary Road, Bangalore, Karnataka 560024 India; 2 Department of Global Health and Population, Harvard T.H. Chan School of Public Health, Boston, 02115 USA; 3 The Institute for Social and Economic Change, Dr. VKRV Rao Road, Teachers Colony, Nagarabhavi, Bangalore, Karnataka 560072 India; 4 Centre for BioSystems Science and Engineering, 3rd Floor, C Wing, Biological Sciences Building, Indian Institute of Science, Bangalore, 560012 India

**Keywords:** Dried blood spots, Whole genome sequencing, Population studies

## Abstract

**Background:**

Dried blood spots (DBS) are a relatively inexpensive source of nucleic acids and are easy to collect, transport, and store in large-scale field surveys, especially in resource-limited settings. However, their performance in whole-genome sequencing (WGS) relative to that of venous blood DNA has not been analyzed for various downstream applications.

**Methods:**

This study compares the WGS performance of DBS paired with venous blood samples collected from 12 subjects.

**Results:**

Results of standard quality checks of coverage, base quality, and mapping quality were found to be near identical between DBS and venous blood. Concordance for single-nucleotide variants, insertions and deletions, and copy number variants was high between these two sample types. Additionally, downstream analyses typical of population-based studies were performed, such as mitochondrial heteroplasmy detection, haplotype analysis, mitochondrial copy number changes, and determination of telomere lengths. The absolute mitochondrial copy number values were higher for DBS than for venous blood, though the trend in sample-to-sample variation was similar between DBS and blood. Telomere length estimates in most DBS samples were on par with those from venous blood.

**Conclusion:**

DBS samples can serve as a robust and feasible alternative to venous blood for studies requiring WGS analysis.

**Supplementary Information:**

The online version contains supplementary material available at 10.1186/s12920-021-00951-w.

## Background

Dried blood spots (DBS) have been used in population-based studies for years to quantify biomarkers and screen for infectious diseases and inherited metabolic disorders in newborns [1–4]. However, because the number of extractable biomolecules is small in DBS, the suitability of this sample type for generating high-quality data for genomic analysis is often debated. While published data from DBS samples using targeted next-generation sequencing (NGS) are available, data from whole-genome sequencing (WGS) are sparse [5, 6].

Most population studies that involve WGS use venous blood (referred to as “blood” hereafter). However, collecting, transporting, and storing blood in field settings present challenges. A trained phlebotomist must collect blood samples in ethylenediaminetetraacetic acid (EDTA) tubes, which are then shipped at a low temperature. These dependencies make blood collection expensive, cumbersome, and logistically unviable for large cohort studies, especially in resource-poor settings.

Given that DBS is inexpensive, scalable, easy to handle in the field and does not require a trained professional for collection [7, 8], we evaluated whether this sample type is suitable for WGS studies. To this end, we assessed the quantity and quality of DNA and WGS data of DBS samples with matched blood in a cohort of 12 subjects collected from the field. We matched the pairwise data for single-nucleotide variants (SNVs), insertions and deletions (InDels), and copy number variants (CNVs). In addition, we compared mitochondrial heteroplasmy variants and copy numbers and telomere lengths in these pairwise samples to evaluate the utility of DBS in population-based studies with end points such as haplogroup analysis, detection of heteroplasmy, and inheritance of mitochondrial diseases. Our results indicate that SNV/InDel concordance is high between DBS and blood, and telomere length estimates are similar between the two sample types. Mitochondrial copy number estimates were found to be matrix-specific, though the sample-to-sample differences were similar. Overall, we conclude that DBS is suitable for WGS analysis and can be used in population studies requiring field-collected samples.

## Methods

### Subjects and study design

Blood samples and matched dried blood spots were collected from 12 residents of Tumakuru district of Karnataka, India, after obtaining requisite permissions and consent. These subjects were chosen to ensure that the selected cohort had a comparable representation of males and females across different age groups (Table 1). The sample collection protocol was independently approved by the ethics committees at Strand Life Sciences and the Institute for Social and Economic Change. The samples were collected in a field setting and shipped to Strand’s laboratory for processing. All the methods were carried out in accordance with relevant guidelines and regulations.

**Table 1:** Age and gender distribution

Age group (years)	Female	Male
< 50	2	2
51–60	2	3
61–70	1	1
> 80	1	0
Total	6	6

### Sample collection and storage

For DBS samples, drops of blood were collected from a needle prick and spotted onto circles on Whatman FTA cards (Sigma-Aldrich, MO, USA). Three to four drops of blood were spotted per circle. The blood spots were air dried, packed, and then shipped to the lab at ambient temperature. DBS samples were stored at room temperature in a dry condition until DNA extraction. Blood samples (5 mL) were collected in K2-EDTA tubes. They were transported to the lab in cool packs (4 °C) within 12 h of collection and were stored at 4 °C until DNA was extracted.

### DNA isolation

DNA was isolated from DBS samples within 40 days of collection using 6–7 punches of 2–3 mm diameter (two FTA card circles). Using the Formapure extraction and purification kit, DNA was manually extracted from pooled DBS punches. DNA was extracted from blood samples within 25 days of collection using the Agencourt Genfind v2 DNA isolation kit (Beckman Coulter, CA, USA), as recommended by the manufacturer, with the automated Biomek4000 (Beckman Coulter, CA, USA).

### Library preparation and sequencing

WGS libraries were prepared using the Nextera DNA Flex Library Prep Kit (Illumina Inc., CA, USA). Input DNA of 100 ng was used for library preparation with minimal cycles of polymerase chain reaction (PCR) as recommended by the manufacturer. Adapter-tagged sequences were inserted into fragments of genomic DNA using bead-based transposons. Unique indices were added to each sample during PCR amplification. The PCR-amplified libraries were then purified and analyzed for size using TapeStation 2200 (Agilent Technologies, CA, USA). Normalized libraries were pooled and sequenced on the Illumina NovaSeq 6000 system using the high-output 150 × 2 base pair S4 flow cell and reagents.

### Bioinformatics quality control

All bioinformatics analyses were performed using Strand NGS ver. 3.3 (Strand Life Sciences, Bangalore, India). Raw reads were mapped to the human genome reference hg19. Distributions of base quality, read quality, mapping quality, insert length, and mapping type (normal, mate far and near, and mate flip) were plotted for both DBS and blood samples.

### Coverage analysis

Coverage analysis was carried out at both the whole genome and whole exome levels. For whole exome coverage, read depth and percentage of bases with low coverage (threshold of 20X) were calculated on the regions of the SureSelect Human All Exon V7 manifest covering 35.8 Mb and 19,634 genes. Further, the correlation between whole-exome region-wise coverage of DBS and blood samples was calculated. In addition, the correlation between whole-exome region-wise coverage of DBS and blood samples was calculated.

To study the equivalence of the DBS and the blood samples for CNV calling, coverage analysis at the whole genome level was carried out. The genome was divided into windows of size 5kbp. For each blood-DBS sample pair, windows with an average coverage of less than 10 × or more than 250 × in the blood sample were ignored. Chromosomes X and Y were ignored for uniformity of analysis across all sample pairs. The read count was normalized by the total number of reads in the sample, and the ratio of normalized read counts in the DBS sample to that in the blood sample was calculated. Windows with the normalized read count ratio < 0.7 or > 1.3 were considered as discrepant. These thresholds were used by Ganapathy et al. (2019) for reliable detection of copy number alterations using Strand NGS [9].

### CNV calling

To detect CNVs, the genome was divided into small windows [10], and read counts were obtained for each of these windows across all samples. Windows with fewer than five and more than 100,000 reads were ignored. For each window, read count ratio was computed for each sample by comparing against all others in the same matrix-specific batch. CNV regions were detected from the ratios by a process called segmentation in which windows are merged into larger segments if they have similar ratios. Two segments were combined only if the distance between them was less than 10% of the total length of the two segments. Segments with amplifications needed to satisfy an additional criterion that the difference in the copy numbers was less than 20% of their mean copy number. The minimum segment size was set to five windows. CNVs were identified in each sample before and after correcting for GC bias (i.e., the effect of GC content on read coverage across a genome).

### SNV/InDel calling

SNVs and InDels were detected in the samples using the germline variant calling method in Strand NGS. SNV/InDel concordance between two samples was calculated as

$$\frac{2C}{{(2C) + U1 + U2}} \times 100$$

where ***C*** is the number of variants common to both samples and ***U1*** and ***U2*** are variants unique to samples 1 and 2 respectively. Concordance was calculated for substitutions and for InDels after excluding regions annotated by RepeatMasker (size: 1448 Mb) from UCSC Genome Browser. Any variant with read depth < 20 or % allele frequency (%AF) < 20% were excluded because they are likely to be false positives. There was < 1% difference (p > 0.05) in the percentage of filtered variants between DBS and blood samples across all the 12 pairs.

### Mitochondrial heteroplasmy, haplotypes, and copy number changes

Raw FASTQ files from both DBS and blood were aligned against the mitochondrial reference genome (Cambridge reference sequence NC_012920.1 using Strand NGS ver. 3.3). Only reads that were > 95% identity matched to the reference were used. Realignment was carried out to improve the alignment of reads that were split because of the circular genome. Uniquely mapping reads were used to calculate the average nuclear genome coverage. The effective genome size of 2,827,437,033 bp was used. The mitochondrial copy number was calculated as [11]

$$\frac{Mitochondrial\;depth\;of\;coverage}{Nuclear\;depth\;of\;coverage}\times2$$

The nuclear coverage was calculated as follows:

$$\frac{No.\;of\;unique\;reads}{{Effective\;genome\;size}} \times 150$$

To estimate heteroplasmic mitochondrial mutations, variant calling at allele frequency (AF) ≥ 2% were performed for SNV identification on the mitochondrial genome (after applying standard filters on read quality, alignment and mapping quality, and strand bias). Heteroplasmic mitochondrial mutations were identified with the following criteria: (a) sequence quality > 20, (b) coverage > 200X, (c) mutant AF between 2 and 90%, and (d) variant present in < 20% of samples. Additionally, for variants between 2 and 5% AF, we ensured that the %AF was at least three standard deviations away from the trimmed mean.

For haplogroup analysis, SNV files were exported from Strand NGS with only mtDNA substitutions and converted to the *hsd* format using custom scripts. The *hsd* files were input to the HaploGrep tool (v2) for haplogroup classification [12, 13]. A phylogenetic tree was constructed using default settings. Clustering analysis was performed on all the DBS and blood samples to assess mitochondrial SNV (mtSNV) concordance and to identify contamination.

### Telomere length estimation

TelSeq software was used to estimate the length of the telomeres in the DBS and blood samples. Aligned BAM files with all reads were used as inputs. Telomere length was calculated as described in Ding et al. [14]. Briefly, the number of telomere signature TTAGGG repeats, GC content of the reads, and a constant based on the length of the genome were used in the calculations. A threshold of seven was applied for the number of repeats in a read, and a GC content window of 48–52% was used for the length estimate, which were the default settings of the tool.

## Results

### Comparison of genomic DNA quality

We extracted genomic DNA (gDNA) from paired DBS and blood samples from 12 subjects. Individual DBS samples consisting of 5–6 punches yielded an average of 311 ng (± 123 ng) of gDNA. Yields from corresponding 200 µl of blood were tenfold higher. Nevertheless, all yields were greater than the 100 ng input recommended for whole-genome library preparation (Table 2). The A260/A280 ratio (an indicator of the purity of nucleic acid samples) values were measured and calculated for gDNA from DBS and blood samples, which were found to be in the reference range of 1.7 to 2. The average size of DNA fragments was 21 kb, for both DBS- and blood-derived DNA. The quality of gDNA extracted from DBS was on par with that of blood.

**Table 2:** Comparison of gDNA extraction QC, library preparation QC metrics

Subject details			DBS							Blood						
ID	Age (yrs)	Sex	A260/280	DNA Yield (µg)	Lib conc (ng/µl)	Lib yield (ng)	Lib avg size (bp)	Yield (Mb)	Average coverage	A260/280	DNA Yield (µg)	Lib conc (ng/µl)	Lib yield (ng)	Lib avg size (bp)	Yield (Mb)	Average coverage
S-10438	70	F	1.700	0.169	11.5	345	598	1,31,491	49.09	1.922	4.52	10.9	327	628	1,44,728	45.50
S-10439	43	F	1.873	0.175	11.0	330	603	1,35,060	48.34	1.913	3.35	11.8	354	627	1,43,382	45.88
S-10440	58	M	2.000	0.195	10.1	303	622	1,32,497	45.15	1.934	4.54	11.0	330	637	1,35,938	45.50
S-10441	60	M	1.830	0.212	10.5	315	606	1,41,057	48.20	1.961	2.19	12.1	363	629	1,44,174	47.40
S-10442	68	M	2.000	0.255	10.6	318	619	1,30,753	44.80	1.984	2.04	10.7	321	627	1,33,614	44.74
S-10444	44	M	1.897	0.490	11.5	345	636	1,44,365	45.16	1.907	4.93	11.2	336	637	1,33,992	50.14
S-10447	85	F	1.738	0.239	11.2	336	608	1,26,642	49.31	1.896	2.94	11.1	333	617	1,45,442	43.40
S-10450	45	F	1.793	0.282	11.3	339	612	1,26,180	45.05	1.899	5.70	12.2	366	611	1,34,580	43.39
S-10452	57	M	1.792	0.234	11.1	333	619	1,54,719	48.81	1.897	5.60	11.8	354	641	1,46,397	53.02
S-10454	52	F	1.991	0.289	11.2	336	599	1,59,173	52.28	1.923	1.94	11.0	330	573	1,54,437	55.00
S-10457	48	M	1.872	0.285	12.2	366	634	1,52,925	44.84	1.895	1.40	10.3	309	663	1,33,573	52.09
S-10458	53	F	1.889	0.534	10.6	318	644	1,09,371	45.49	1.902	3.79	11	330	566	1,35,132	38.09

### Comparison of sequencing metrics

**Fig. 1:**
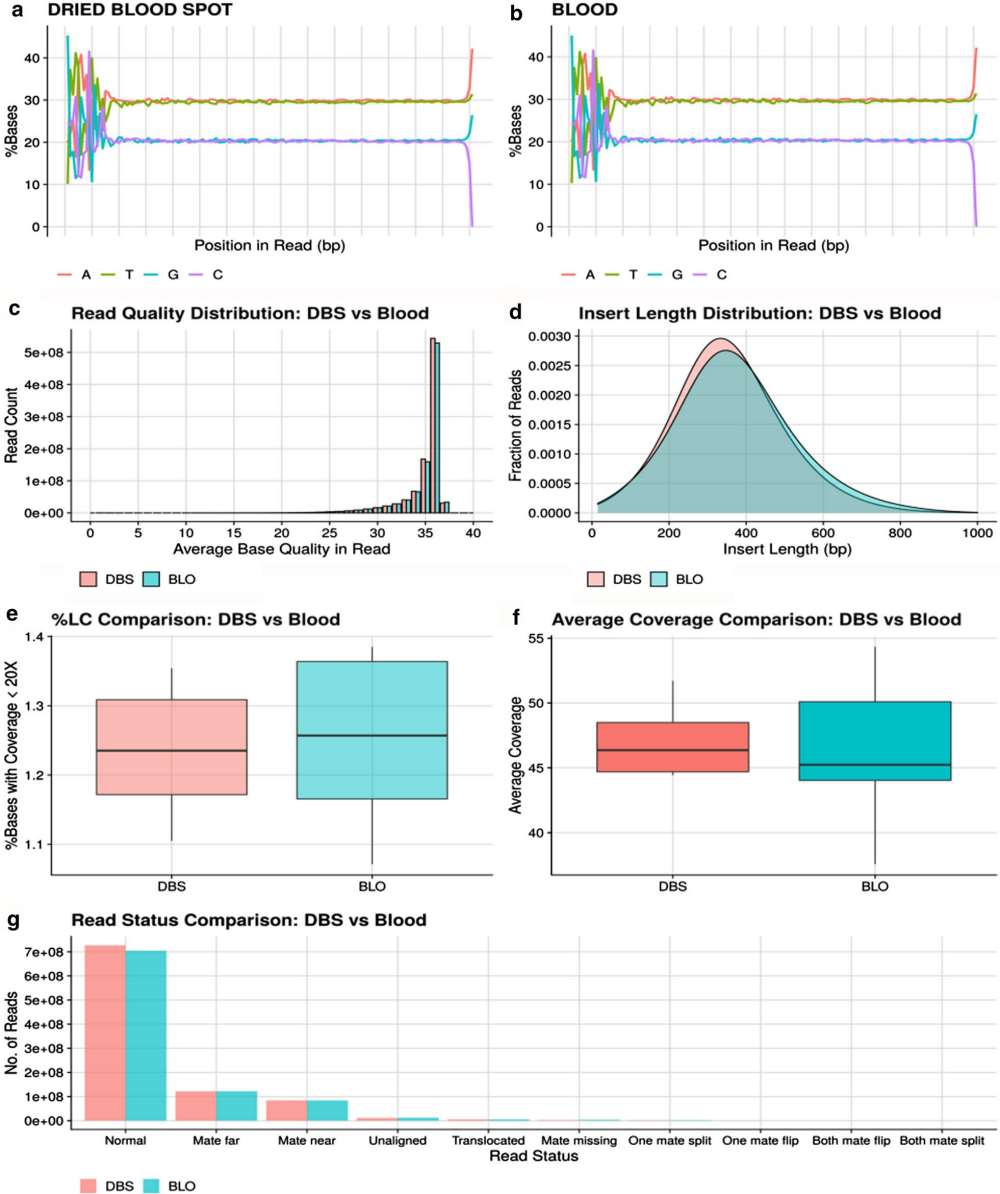
Comparison of sequencing QC between DBS and blood. Base composition by position in reads in **a** DBS samples and **b** blood samples; **c** read quality distribution in DBS and blood; **d** insert length distributions in DBS and blood; **e** distribution of % of low, covered bases (< 20X) across DBS and blood samples; **f** distribution of average coverage in DBS and blood; and **g** bar chart of the number of reads based on read alignment status

WGS libraries were prepared using all samples and showed similar yield and size (Table 2). All sample libraries were pooled and run on a NovaSeq S4 kit. The samples had an average of 140 GB data and > 0.8 billion reads except for a single blood sample. No significant differences in the sequencing quality control (QC) metrics were evident between DBS and blood samples. QC plots comparing base and read quality distributions, read fractions uniquely mapped to the genome, and read types (such as normal, mate far, mate near, etc.) were very similar for both matrices (Fig. 1a–c, Fig. 1g). Insert lengths were marginally smaller in DBS samples when compared with blood (10–15 bp) (Fig. 1d) but were fairly uniform across all samples within each matrix type.

### Comparison of coverage

Coverage analysis was performed at both exome and genome levels. For whole-genome level coverage analysis, the Pearson correlation coefficient computed between the normalized coverage in the DBS and the blood samples was very high for all the pairs (R^2^ > 0.97). Also, the mean of the normalized coverage ratio between the paired samples, as expected, was close to 1 while the standard deviation was in the range of 0.05–0.08. In addition, windows for which the ratio was < 0.7 or > 1.3 were marked as discrepant. The percentage of such discrepant windows was very low (< 0.035%) for all the pairs, indicating excellent coverage concordance between the two matrices.

In the exome-level analysis, approximately 12% of the reads mapped to exons and were within 1000 bp of the exonic boundaries; the results were similar for DBS and blood samples. Figure 1e, f shows the average coverage and the percentage of under-covered bases with respect to the whole exome for the 12 pairs of samples. Average coverage is in the range of 40 to 55X and the percentage of undercovered bases (i.e., bases with coverage < 20X) is between 1% and 1.5% for both sample types. Correlation coefficients of average coverage per exon between each pair of samples ranged from 0.82 to 0.90. Given that copy number calling is dependent on depth of coverage (DoC) and DoC in DBS is comparable to that of blood samples at the whole-genome and gene levels, copy number calling in DBS samples would be an acceptable surrogate for the same in blood samples. As an example of detecting a clinical-grade CNV, a 1.1Mbp amplification was detected in both DBS and blood samples from one subject (Additional file 1: Figure S1).

### SNV/InDel concordance

SNVs and InDels were called in all 12 pairs of samples and analyzed for concordance. Concordance was evaluated for single-base substitutions and InDels separately in each of the 12 pairs before and after masking repeat regions. As a measure of DNA integrity [15], the transition/transversion ratios were calculated for all 12 pairs and were found to be approximately 2 for all cases, the ideal for homologous strands of DNA. This indicated no biased damage in the DBS gDNA. Overall concordance was > 99% for substitutions and > 91% for InDels (Table 3). The number of substitutions and InDels are in the expected ranges of 3–4 million and 200 k–400 k respectively in both matrices [16]. Paired sample t-tests on these metrics did not show any statistically significant difference (p > 0.05) between the two matrices. Thus, DBS provides high-quality data for WGS analysis on par with blood.

**Table 3:** Comparison of SNVs / InDels

Subject details			#Substitutions		#Insertions		#Deletions		Concordance		
ID	Age (year)	Sex	DBS	Blood	DBS	Blood	DBS	Blood	Substitutions (%)	Insertions (%)	Deletions (%)
S-10438	70	F	3,662,236	3,637,951	170,193	168,427	194,060	192,420	99.09	92.11	92.37
S-10439	43	F	3,677,398	3,658,769	170,770	169,156	194,380	193,432	99.13	92.35	92.44
S-10440	58	M	3,600,908	3,589,227	165,298	165,334	189,077	188,536	99.11	92.25	92.35
S-10441	60	M	3,622,018	3,613,559	167,551	167,124	190,497	190,118	99.07	92.38	92.54
S-10442	68	M	3,517,494	3,505,755	162,815	162,246	186,736	185,511	99.11	92.05	92.32
S-10444	44	M	3,581,795	3,604,133	165,715	167,838	188,091	189,228	99.12	92.38	92.61
S-10447	85	F	3,654,924	3,615,037	170,261	167,953	193,700	191,085	99.14	92.34	92.53
S-10450	45	F	3,660,609	3,634,748	169,833	168,578	193,626	191,662	99.13	92.19	92.35
S-10452	57	M	3,615,461	3,629,755	167,588	168,783	190,852	191,136	99.14	92.40	92.73
S-10454	52	F	3,697,756	3,702,642	172,643	172,321	195,143	194,984	99.16	92.52	92.79
S-10457	48	M	3,569,188	3,610,732	164,799	168,364	188,391	190,266	99.13	92.31	92.68
S-10458	53	F	3,650,381	3,507,492	168,600	159,080	193,819	183,910	99.11	91.94	91.95
One-tailed p-value from paired sample t-test (at alpha = 0.05)			0.12		0.17		0.07		–		

### Downstream analysis of WGS data

WGS data have also been used in more complex analyses such as (a) haplotyping for ancestry determination, (b) estimating mitochondrial heteroplasmy in a population to understand inheritance of rare mitochondrial disorders, (c) estimating mitochondrial copy numbers, and (d) estimating telomere length as surrogate biomarkers for aging or age-related disorders. For these analyses, assessing whether WGS data from DBS would match the data obtained from blood is critical.

#### Haplogroup assignment

Both DBS and blood samples paired into individual clades, indicating a high concordance of mtSNVs between the two matrices (Additional file 2: Figure S2). Nine subjects were found to belong to the haplogroup expected for the population of the Indian subcontinent. The remaining three subjects mapped to the H haplogroup (Europe and Central Asia). Findings of H haplogroups (H5a1, H6a1a, and HV6) have been reported among South Indian populations [17–20].

#### Heteroplasmy analysis

Table 4 shows that 11 heteroplasmy variants were found across seven subjects (58% of the cohort) with further details in Additional file 3: Table S1. Four subjects had one heteroplasmy, two subjects had two, and one subject had three. Interestingly, six subjects (86%) with observed heteroplasmy were older than 50 years of age (67% of the cohort is > 50). The same variants were observed at similar frequencies in both DBS and blood samples.

**Table 4:** Mitochondrial copy number, haplogroup, and heteroplasmy

ID	Gender	Age	DBS MtCN	Blood MtCN	Mt haplogroup	No. of heteroplasmies	DBS telomere length (kbp)	Blood telomere length (kbp)
S-10439	F	43	368.46	197.27	H5a1	0	3.19	3.18
S-10450	F	45	350.90	185.80	M36b	0	3.17	3.47
S-10457	M	48	263.46	207.09	M5a2a2	0	3.13	2.81
S-10454	F	52	280.45	193.03	R30b2a	1	3.19	3.06
S-10458	F	53	232.34	107.99	H6a1a	2	3.17	2.77
S-10452	M	57	230.74	211.94	M5a2a1	0	2.99	3.20
S-10440	M	58	212.65	126.89	M39b	3	3.47	2.51
S-10441	M	60	223.17	139.76	M6a1a	0	3.19	2.73
S-10442	M	68	234.91	147.58	M3a1 + 204	1	3.44	3.15
S-10438	F	70	330.57	234.10	M35a	1	3.20	3.28
S-10447	F	85	239.21	144.04	M2a1	2	3.28	2.79
S-10444	M	44	217.63	132.80	HV6	1	3.36	3.03

*MtCN* mitochondrial copy number

#### Mitochondrial copy number changes

The average mitochondrial copy number (MtCN) from the blood samples was 169 (± 40), which matches the numbers reported in the literature [21] (Table 4). DBS samples had a higher average MtCN of 266 (± 54). A 0.65 coefficient of correlation (positive trend) is seen between the MtCN reported across both matrices. A slight negative trend in MtCN was observed with respect to age in the DBS samples [22].

#### Telomere length estimation

**Fig. 2:**
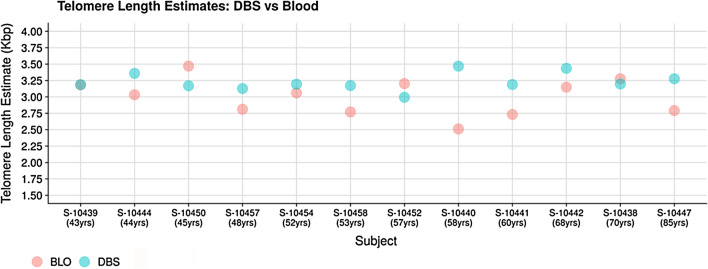
Telomere length comparison between DBS and blood. The telomere length estimates from DBS and blood appear to be similar in most subjects, indicating that DBS could potentially be used for telomere length estimation

Telomeres are believed to shorten with every round of cell division and undergo age-dependent attrition. Analyzing their length is an important component of studies that investigate aging and age-related disorders. Ding et al. [14] developed a novel method called TelSeq to measure average telomere length from the whole genome and exome shotgun sequencing data. This approach measures the number of copies of hexameric repeats (TTAGGG or CCCTAA) in the NGS reads. An important observation was that nine of 12 DBS sample estimates were within 10% of the corresponding estimates from blood samples (Fig. 2). Two of the remaining three were close to 15% of blood. Indeed, the median telomere length in DBS was 3.19 (± 0.13) as compared to 3.04 (± 0.28) in blood. However, our sample size was too small to determine any significant relationship between age and telomere length. Our results show that measurements of telomere length from DBS will yield similar results to those from blood.

## Discussion

DBS-based sampling and archiving of DNA offer a cost-effective, minimally invasive sample collection method that does not require trained phlebotomists or expensive cold chain shipment [7, 8]. In this study, we explored whether DBS-based collection would facilitate genetic and genomic analysis in epidemiological and population-based studies that involve field collection of specimens. While previous NGS studies have compared DBS DNA libraries with matched blood DNA libraries for targeted regions of the genome [5], comprehensive downstream analysis of whole-genome sequencing is pending. In this study, we compared WGS data from matched DBS and blood samples collected from 12 individuals. The goal was to systematically compare and document all quality parameters and data from both sample matrices for WGS analysis. Our cohort was chosen to include an equal number of male and female subjects with ages ranging from 40 to 85 years to specifically address challenges in downstream analyses relevant to aging studies and age-related disorders.

Our results show close to identical performance in the yield and profiles of the NGS libraries from both DBS and blood. While the DNA yield from two saturated DBS spots (5–6 punches) was one-tenth (~ 300–400 ng) of the quantity of DNA obtained from 200 µl of venous blood, the quantity from both sample types was sufficient for downstream library preparation. The DBS libraries showed no loss of diversity of molecules at this level of DNA yield. Quality parameters such as coverage, base quality, read mapping quality and total number of SNVs were similar in both matrices. These sequencing metrics were within acceptable ranges as known from literature on WGS using blood [5]. DNA from DBS samples may be slightly more nicked than blood because we observed that DBS library inserts were roughly 10–15 bp shorter. However, given that DBS-derived libraries [5, 6] had a similar depth of coverage as that of matched blood, any compromise in molecular diversity affecting downstream analysis can be ruled out. More importantly, all 12 samples had a tight distribution for yield and quality, indicating high reproducibility of data between DBS and blood. SNVs, InDels, and CNVs detected in DBS and blood also showed excellent concordance.

We then evaluated the quality of DBS-derived data for several downstream analyses often used in research pertaining to ancestry, aging, and age-related disorders. These analyses included determining haplogroups and estimating mitochondrial heteroplasmy, mitochondrial copy number changes, and telomere length shortening. Mitochondrial SNV-based haplogroup and heteroplasmy analyses on DBS- and blood-derived DNA gave identical results. As expected, the haplogroup assignments revealed that 75% of the subjects belonged to the M subgroup, which is predominant in the Indian subcontinent. The remaining subjects were part of the H haplogroup, which has also been previously reported in the Indian population.

Mitochondrial heteroplasmy, a parameter analyzed in the context of age-related disorders, was identified mostly in subjects above 50 years of age. We observed that the heteroplasmy variants identified in DBS agreed with those found in blood. In the case of mitochondrial CNV analysis, absolute copy number estimates were higher in DBS data; similar observation is reported by Anderson et al. [23]. However, the trends were similar across matrix-specific samples. While the technical reasons that give rise to the differences in these estimates are unclear, this could be attributed to either the number of platelets captured in the sample preparations [24, 25], the differences in the extraction methods of the two sample types, or the smaller insert size of DBS-derived NGS libraries.

Telomere lengths were comparable between DBS and blood: in nine of the 12 samples, estimates from DBS were within 10% of those from venous blood. Effects of age-related shortening on telomere lengths were not observed in our data. To identify definitive trends, a larger cohort is required with sufficient subjects in each age group. Interestingly, less variance in telomere lengths was observed in the DBS data. Ding et al. [14] show that increased coverage reduces variability in telomere length estimation by TelSeq. DBS data show that coverage is generally higher and more uniform than from blood. The aforementioned analyses may require further analysis from large cohort studies using DBS.

## Conclusions

In summary, DBS provides excellent WGS data for genome-wide SNV, InDel, and copy number analyses. Haplotype analysis and heteroplasmy calculations were on par with venous blood samples. Given the ease of sample collection, transport, and storage, DBS is a robust sample type for genomic analysis in large population-based studies.

## Supplementary Information

**Additional file 1:** Copy number alteration in a blood and DBS sample pair. A 1.1Mbp amplification identified in the blood sample of subject, S-10438, was also identified in the matched DBS sample. The figure shows elevated normalized coverage ratios of each region in the 1.1Mbp stretch for the sample of interest when compared to the remaining samples.

**Additional file 2:** Assignment of matched blood-DBS samples to mitochondrial haplogroups. Mitochondrial SNVs identified in each sample were used to assign them to haplogroups. The cladogram in the figure shows that each pair of matched blood and DBS are assigned to the individual clades thus demonstrating high concordance between blood and DBS samples.

**Additional file 3:** List of heteroplasmy variants identified in subjects. The table lists the mitochondria heteroplasmy variants shortlisted in blood and DBS samples along with the %allele frequencies.

## Data Availability

The datasets generated and/or analyzed during the current study are not publicly available due to subject confidentiality. Sequencing data (not subject identification information) are available upon request from Strand Life Sciences (contact via email to shanmukh@strandls.com or chandru@strandls.com) for researchers who meet the criteria for access to sequencing data.

## Abbreviations

WGS: Whole-genome sequencing
NGS: Next-generation sequencing
DBS: Dried blood spots
SNV: Single nucleotide variant
InDels: Insertions and deletions
