# Supplementary figures and images for: Validation of whole genome sequencing from dried blood spots
Source: BMC Med Genomics. 2021 Apr 20;14:110. doi: 10.1186/s12920-021-00951-w (PMC8056537; doi:10.1186/s12920-021-00951-w)

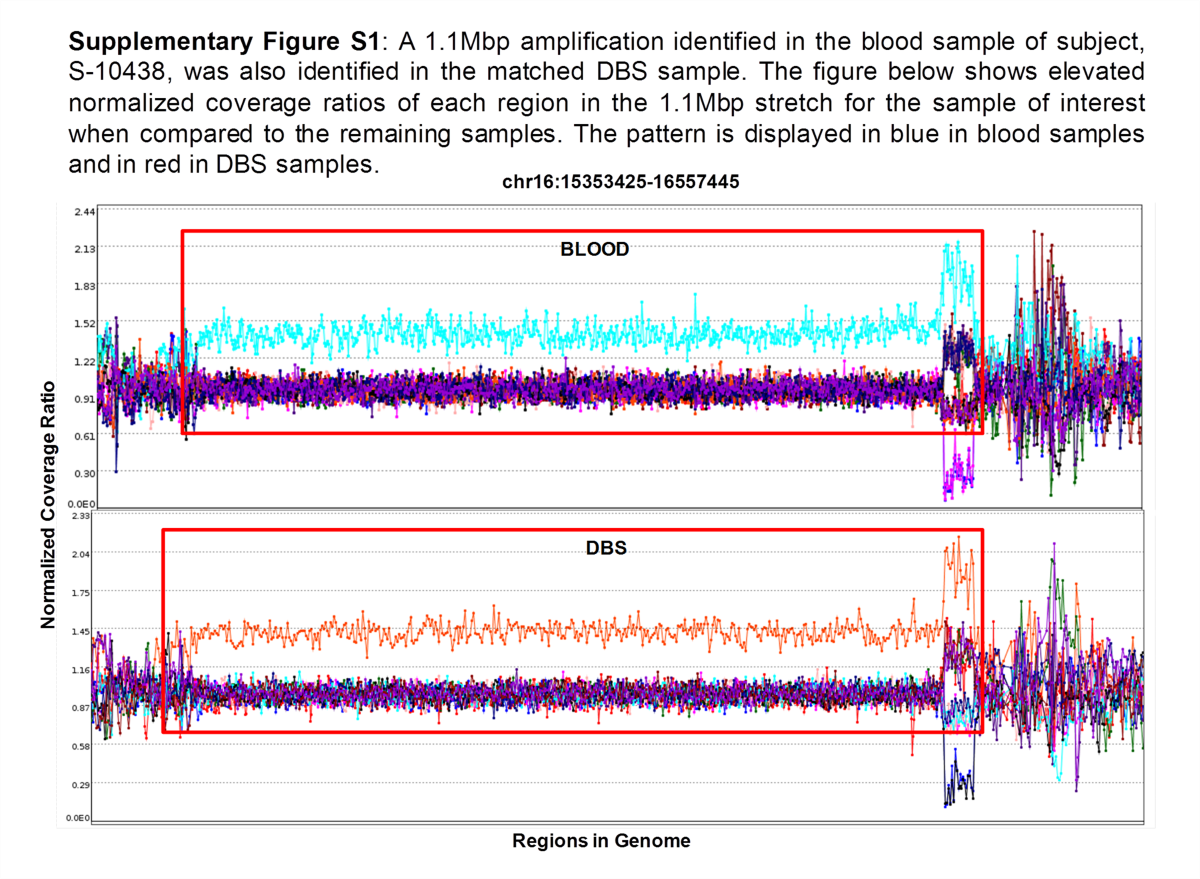

Supplement: Supplementary file 1 — Additional file 1: Copy number alteration in a blood and DBS sample pair. A 1.1Mbp amplification identified in the blood sample of subject, S-10438, was also identified in the matched DBS sample. The figure shows elevated normalized coverage ratios of each region in the 1.1Mbp stretch for the sample of interest when compared to the remaining samples. [file 12920_2021_951_MOESM1_ESM.tiff]

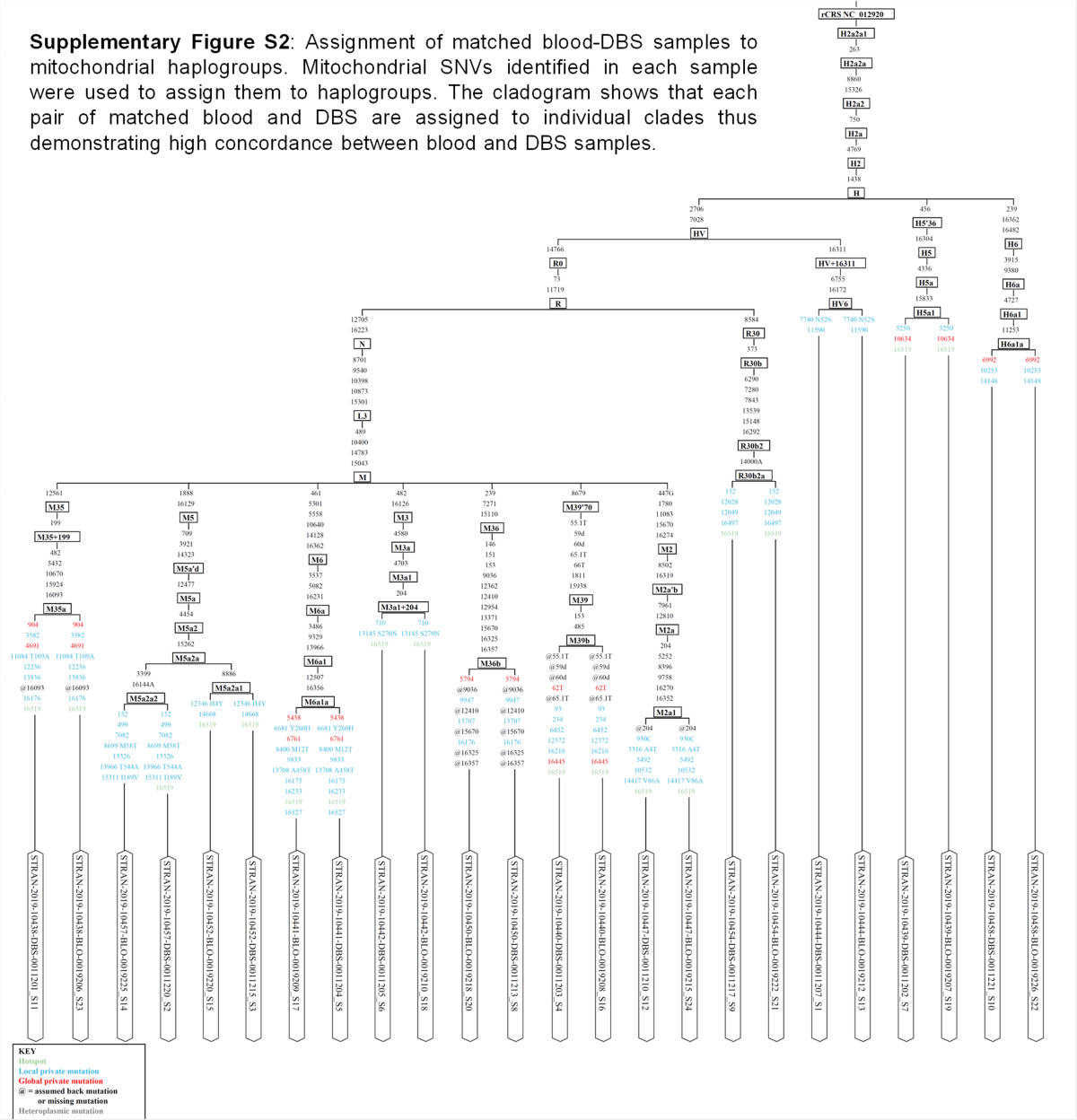

Supplement: Supplementary file 2 — Additional file 2: Assignment of matched blood-DBS samples to mitochondrial haplogroups. Mitochondrial SNVs identified in each sample were used to assign them to haplogroups. The cladogram in the figure shows that each pair of matched blood and DBS are assigned to the individual clades thus demonstrating high concordance between blood and DBS samples. [file 12920_2021_951_MOESM2_ESM.tiff]
