# Supplementary material for: Validation of whole genome sequencing from dried blood spots
Source: BMC Med Genomics. 2021 Apr 20;14:110. doi: 10.1186/s12920-021-00951-w (PMC8056537; doi:10.1186/s12920-021-00951-w)
Supplement: Supplementary file 3 — Additional file 3: List of heteroplasmy variants identified in subjects. The table lists the mitochondria heteroplasmy variants shortlisted in blood and DBS samples along with the %allele frequencies. [file 12920_2021_951_MOESM3_ESM.pdf]

Supplemental Table S1: List of heteroplasmy variants identified in subjects

SAMPLE	HETEROPLASMY	%AF - BLOOD	%AF - DBS
S-10438	chrM:16065G>A	16.76	16.52
S-10440	chrM:656T>C	2.40	3.00
S-10440	chrM:1409A>G	2.59	2.71
S-10440	chrM:12291T>C	6.74	8.20
S-10442	chrM:3666G>A	5.69	5.12
S-10444	chrM:514insCA	2.18	2.25
S-10447	chrM:514insCA	2.32	2.84
S-10447	chrM:3504T>C	2.80	2.89
S-10454	chrM:16189T>C	83.87	83.71
S-10458	chrM:13723T>C	9.09	8.32
S-10458	chrM:14259G>A	10.91	11.07
